# Physiopathological Roles of White Adiposity and Gut Functions in Neuroinflammation

**DOI:** 10.3390/ijms252111741

**Published:** 2024-10-31

**Authors:** Eduardo Spinedi, Guillermo Horacio Docena

**Affiliations:** 1 Centro de Endocrinología Experimental y Aplicada (CENEXA-UNLP-CONICET-CICPBA), University of La Plata Medical School, La Plata 1900, Argentina; 2 Instituto de Estudios Inmunológicos y Fisiopatológicos (IIFP-UNLP-CONICET-CICPBA), School of Sciences, University of La Plata, La Plata 1900, Argentina

**Keywords:** neuroinflammation, hypertrophic white adiposity, insulin resistance, diabetes mellitus, neurodegenerative diseases, central and autonomic nervous systems, immune system, gut–brain axis, microbiota, dysbiosis, brain–WAT–gut axis

## Abstract

White adipose tissue (WAT) and the gut are involved in the development of neuroinflammation when an organism detects any kind of injury, thereby triggering metainflammation. In fact, the autonomous nervous system innervates both tissues, although the complex role played by the integrated sympathetic, parasympathetic, and enteric nervous system functions have not been fully elucidated. Our aims were to investigate the participation of inflamed WAT and the gut in neuroinflammation. Firstly, we conducted an analysis into how inflamed peripheral WAT plays a key role in the triggering of metainflammation. Indeed, this included the impact of the development of local insulin resistance and its metabolic consequences, a serious hypothalamic dysfunction that promotes neurodegeneration. Then, we analyzed the gut–brain axis dysfunction involved in neuroinflammation by examining cell interactions, soluble factors, the sensing of microbes, and the role of dysbiosis-related mechanisms (intestinal microbiota and mucosal barriers) affecting brain functions. Finally, we targeted the physiological crosstalk between cells of the brain–WAT–gut axis that restores normal tissue homeostasis after injury. We concluded the following: because any injury can result not only in overall insulin resistance and dysbiosis, which in turn can impact upon the brain, but that a high-risk of the development of neuroinflammation-induced neurodegenerative disorder can also be triggered. Thus, it is imperative to avoid early metainflammation by applying appropriate preventive (e.g., lifestyle and diet) or pharmacological treatments to cope with allostasis and thus promote health homeostasis.

## 1. Introduction

Neuroinflammation is an inflammatory response that disrupts central nervous system (CNS) homeostasis; it can be activated by broad etiologies, e.g., infection, traumatic brain injury, toxic metabolites, malnutrition, and autoimmunity. Neuroinflammation may take place at any point in the human lifetime, with serious health consequences ranging from metainflammation (obesity-driven metabolic dysfunction and chronic low-grade inflammation) to neurodegenerative diseases (NDs). Neuroinflammation is known to involve disruption in signaling pathway proteins, receptor activities, and a variety of cell functions.

Moreover, the involvement of several key components is related to the development and maintenance of neuroinflammation, e.g., epigenetic markers, activated microglia, infiltrating T cells, matrix metalloproteinases (MMPs), ion channels, microRNAs, insulin receptors, and the autophagic process [1]. In fact, any kind of peripheral inflammation triggers neuroinflammation, wherein blood–brain barrier (BBB), glia, and neuron dysfunctions are involved [2]. This neuro-immune crosstalk is not one-way but rather a complex, bidirectional system, wherein the CNS and the immune system constantly communicate and influence each other. Indeed, when the BBB is compromised, harmful substances enter the brain, modifying its undoubtedly delicate environment. After these substances enter the brain, brain-resident homeostatic microglia cells are activated, and local inflammation is consequently triggered. Symptoms of neuroinflammation may arise, such as slow metabolism, body weight gain, diabetes mellitus, dysbiosis, and mood and neurodegenerative disorders [1,3]. Moreover, neuroinflammation is undoubtedly involved in the physiopathology of serious degenerative diseases (NDs) [e.g., Parkinson’s disease (PD), Alzheimer’s disease (AD), Huntington’s disease (HD), and amyotrophic lateral sclerosis (ALS)] [1]. “Activated” microglia cells and elevated pro-inflammatory factors are clearly associated with CNS function damage. Microglia comprise immune cells in the CNS and play a key role in maintaining their homeostasis [3]. Astrocytes control blood flow and extracellular neurotransmitter levels to ensure that their endogenous microenvironment is optimal for neuron function. Without stimulation or injury, neurotransmitters, neurotrophic factors, anti-inflammatory cytokines, and intercellular contacts inhibit neuroglia activity [4,5]. However, when the brain’s environment is disrupted, microglia cells are stimulated and secrete an excess of pro-inflammatory factors. The brain compartment is highly preserved by an active immune function in physiological conditions. In fact, antigen presentation is actively inhibited, microglia cells are maintained as “homeostatic”, and immune components are excluded from the brain by the BBB. However, once brain injury occurs, “activated” microglial cells produce pro-inflammatory factors, thereby inducing neuroinflammation. Whether microglia elicit detrimental or beneficial effects in brain neurons is dependent on the transition from their “homeostatic” to “activated” state [6,7].

This review centers on the pivotal role of injured tissues (WAT and the gut) in promoting neuroinflammation. It delves into how these endogenous responses impact both local and distant tissues and how the brain’s defense mechanisms are activated to restore homeostasis. The mechanistic and pathological observations presented here provide a deeper understanding of the consequences of WAT and gut participation in the neuroinflammatory process.

## 2. Relevance of WAT and Gastrointestinal Tract Functions in Neuroinflammation

White adipose tissue and the gut, as metabolic–endocrine organs, closely interact both peripherally and through the CNS. Because this interaction, which is facilitated by the blood and the vagus nerve, has significant implications on the physiology of the CNS [8], it has been coined the WAT–gut axis [9]. The CNS, in response, plays a crucial role in enhancing the production and secretion of brain-derived neurotransmitters and neuropeptides, activating various hormone axes and, through autonomous nervous system (ANS)-dependent function, regulating several peripheral mechanisms, including those of WAT, the gastrointestinal tract, and the immune system [10]. The dynamic loop between the CNS and the WAT–gut axis is not only vital for survival in both physiological and pathological conditions but is also fascinating in its complexity and potential for further research.

The functions of WAT and the gastrointestinal tract are highly relevant in the context of neuroinflammation. The BBB plays a key role in neuroinflammation. Indeed, changes in the BBB’s permeability [11] and function after systemic inflammation due to increased peripheral pro-inflammatory signals in blood have been reported. In turn, messages entering the brain, originated by various molecules and pathogens, cause brain neuroinflammation and likely, if it is sustained, neurodegeneration [12]. More recently, a gut vascular barrier (GVB) was described [13], as was a connection between the BBB and the GVB that may interfere with cognitive behavior [14]. The GVB is composed of interacting endothelial cells, glial cells, and pericytes; this barrier prevents the translocation of large molecules from the gut lumen [15].

When excessive WAT (namely, the visceral–mesenteric depot) accumulation occurs, such as in different obese phenotypes, tissue dysfunctionality can activate immune cells, resulting in a chronic degree of peripheral inflammation [16]. In fact, obesity is associated with neuroendocrine, metabolic, and immune system disorders such as the enhancement of inflammatory cytokine production and release, overall insulin resistance (IR), vascular endothelial inflammation, and atherosclerosis, among others [10]. Consequently, the development of a deep dysmetabolic phenotype increases the individual’s susceptibility to the further development of type 2 diabetes mellitus (T2DM).

The microbiota is also a key player in this loop, and interactions between intestinal microbes with immune cells and neurons has been extensively described to shape the physiology of intestinal and distant tissues in both homeostatic and pathological circumstances. Altered gut microbiota (GM) has been claimed to be critical in tumor development, allergy, and other inflammatory disorders. However, the mechanisms whereby it occurs remain a matter of study [17]. In general, inflammatory bowel diseases (IBDs) are disorders associated, with obesity, among other factors, playing a role in their development and course [16]. For instance, in Crohn’s disease, a crucial role of mesenteric WAT in the pathophysiology of intestinal inflammation has been reported [18]. The involvement of large-size mesenteric adipocytes producing an excess of pro-inflammatory adipokines (e.g., leptin, TNF-α, PAI-1, and resistin) is implicated in the pathogenesis of IBDs [16]. As a result, an excess of WAT-secreted pro-inflammatory adipokines and pro-inflammatory cytokines (e.g., TNF-α, IL-1, IL-6, IFN-γ) in the gut impacts systemically (through peripheral blood) and in the ANS, on the hypothalamus, inducing several CNS dysfunctions, such as those that occur in neurodegenerative disorders (NDs) [19]. It has been reported that in AD, developed brain IR enhances CNS TNF-α and endothelin-1 production, and IRS-1 signaling deficiency and reduces nitric oxide (NO) production. As a result, low brain blood flow and increased neuroinflammation characterize AD patients [20].

The main function of the gastrointestinal tract is food processing, digestion, and absorption of nutrients. To accomplish this complex task, many microbes that probably outnumber the quantity of cells in our body colonize the whole tract. To control this scenario, the organism coordinates the generation of nutrients, absorption and secretion of metabolites, gut motility, and secretion of enzymes and inflammation promoted by the continuous antigenic challenge in the extended intestinal mucosa. To deal with an environment of permanent exposure to different and dynamic stimuli (food components and environmental agents and microorganisms), the tissue must constantly adapt to maintain homeostasis. To achieve this end, the gastrointestinal tract is equipped with a highly specialized mucosal immune system in close contact with a dense network of neurons. The intricate interplay between the nervous and immune systems has evolved as an integrated network that continuously surveys the organism, tracking for internal and environmental stimuli that could disturb tissue homeostasis. This amalgamated circuitry communicates the peripheral tissues with the CNS to respond in different circumstances and play a substantial role in tissue physiology. Inflammation is the hallmark of immune response to harmful stimuli and acts to remove harmful substances, which is followed by the healing process. The nervous and immune systems conduct sensory and effector mechanisms to maintain tissue homeostasis.

Sensing potential pathogenic or harmless stimuli is pivotal to the immune and nervous systems. It promotes a reaction when a microorganism or damaging perturbation is detected in the context of an infection. A delicate and regulated network of immune and neuron cells colocalize in multiple tissues, e.g., mucosal barriers or barrier tissues (skin, lung, and gut), secondary lymphoid organs, and adipose tissue, and this orchestrates host defense mechanisms, thereby restoring tissue physiology. This neuro-immune unit is an integrated circuit that exerts concerted actions in health and disease [21].

The complex neuro-immune crosstalk is bidirectional and relies on cell–cell contacts (direct communication) and soluble factors (indirect communication) that exert modulatory functions on proximal and distant cells. Neurons and immune cells express many common receptors for conserved and vital ligands on microbes (named pattern-recognition receptors or PRR) and cytokine receptors to modulate cell activity. In addition, immune cells express receptors for neurotransmitters and neuropeptides secreted by neurons [22]. Neuro-immune units (anatomical locations wherein immune and neuronal cells colocalize and interact among them to direct tissue physiology) continuously sense peripheral internal and environmental stimuli and communicate the presence of noxious signals to the CNS. Then, immune cells are instructed by the CNS to respond to the tissue receiving the injury and thereafter to promote effective immunity and tissue homeostasis.

### Organization of the Nervous System in the Gut

**Figure 1:**
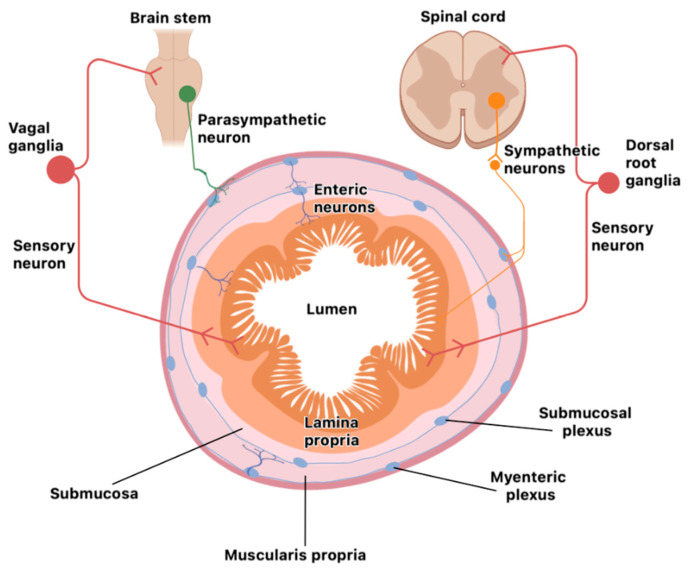
Innervation of the gastrointestinal tract by autonomous neurons. The extrinsic sympathetic neurons (orange) originate from the spinal cord and sympathetic ganglia; the extrinsic parasympathetic neurons (green) originate from the brain stem; and the enteric intrinsic neurons (blue) are distributed in the submucosa and myenteric plexus as a ganglionated circuit. All layers of the intestinal wall are innervated and interconnected. Extrinsic sensory neurons (red) transduce signals from the gut to the brain stem and spinal cord.

The neuro-immune unit can operate in the CNS (brain and spinal cord) or the peripheral nervous system (somatosensory and autonomic nervous systems). Neurons and glial cells in the CNS send efferent signals to peripheral tissues via neurotransmitters and neuropeptides, and immune cells secrete cytokines that bind neuron receptors, either locally or systemically, in the CNS. Some nerves can also signal information back from the tissue to the CNS and then, through efferent nerves, instruct peripheral immune cells for an effective immune response. The vagus nerve is one of the twelve cranial nerves representing the main extrinsic parasympathetic nerve connecting the brain and the proximal colon. It is mainly implicated in modulating the intestinal immune response, depending on the composition of the intestinal microbiota or gut inflammation; the brain promotes, through vagal efferent function, a regulatory signal that controls pro-inflammatory cytokine release. Distal colon parasympathetic innervation originates from the sacral spinal nerves, and both nerves coordinately act as a network [23]. The mucosa and secondary lymphoid organs are highly innervated by the ANS, which is composed of the sympathetic nervous system (SNS), parasympathetic nervous system (PNS), and enteric nervous system (ENS). Cell bodies of the SNS (spinal cord) and PNS (brain and sacral spinal cord) are localized in spinal cord ganglia (extrinsic intestinal neurons). In contrast, those from the ENS are entirely contained within the gastrointestinal tract wall (intrinsic intestinal neurons) and are organized in several plexuses throughout the entire intestinal wall (mucosa, submucosa, and muscle layer). The ENS is the most significant neuron accumulation outside the CNS; it is organized into the myenteric plexus (between the circular and longitude muscles) and submucosal plexus [24] (Figure 1). The ENS comprises sensory neurons that sense intestinal content and motor neurons that drive secretory functions and peristalsis [25].

It is also interesting that the intestine harbors the body’s largest lymphoid cell compartment, concentrated mainly in the mucosa layer that lines with the intestinal lumen [26,27]. This neuro-immune unit allows for a more integrated and concerted immune response, and it seems to be evolutionarily conserved, since non-mammalian organisms (zebrafish, *Caenorhabditis elegans*, Drosophila, etc.) also have integrated mechanisms for protection [28,29].

In conclusion, the neuron and immune systems are actively integrated in the brain–gut axis, and they trigger neuroinflammation upon exposure to harmful stimuli and restore homeostasis once the injury has been controlled.

## 3. Hypertrophic White-Adiposity-Induced Insulin Resistance (IR) as a Main Instigator of Neuroinflammation

### 3.1. Sympathetic Innervation of Adipose Tissue

Although there is controversy regarding the ANS innervation of WAT pad depots, sensory fibers seem to play a relevant regulatory role in WAT functionality. Indeed, as is already accepted, WAT adipocytes are in contact with nerve fibers in the parenchyma [30].

Evidence has indicated that visceral WAT bidirectionally communicates with the brain through afferent sensory fibers and efferent sympathetic fibers [31,32]. The long-isoform of leptin receptor (Ob-Rb) was found on dorsal root ganglia neurons from WAT, thereby suggesting that leptin potentially communicates with sensory nerves in WAT through the dorsal root ganglia [33]. Furthermore, SNS-stimulated lipolysis and intra-WAT injection of free fatty acids can increase WAT afferent nerve activity [34]. Although it was largely hypothesized that these nerves were of an SNS origin, irrefutable proof of SNS innervation of WAT came from a study by Youngstrom and Bartness demonstrating the bidirectional innervation of WAT [35], determining that the SNS ganglia, at T13/L2-L3, innervate WAT pads directly upon adipocytes [36]. In addition, a surgical denervation of WAT depots indicated that sympathetic neurons are synaptically connected and generate neuronal pathways from WAT to the brain [37]. Sympathetic denervation of WAT was also proven to increase the mass depot due to hyperplasia and decreased lipolysis [38]. Conversely, sensory denervation of visceral WAT increased pad mass throughout adipocyte hypertrophy, thereby providing differential ANS control of WAT function among sympathetic and sensory nerves [39,40].

### 3.2. Insulin-Resistant WAT Cells and Metainflammation Precede Neuroinflammation Development

#### Visceral Fat Depots of Large-Size WAT Adipocytes Characterize Obese Phenotypes

**Figure 2:**
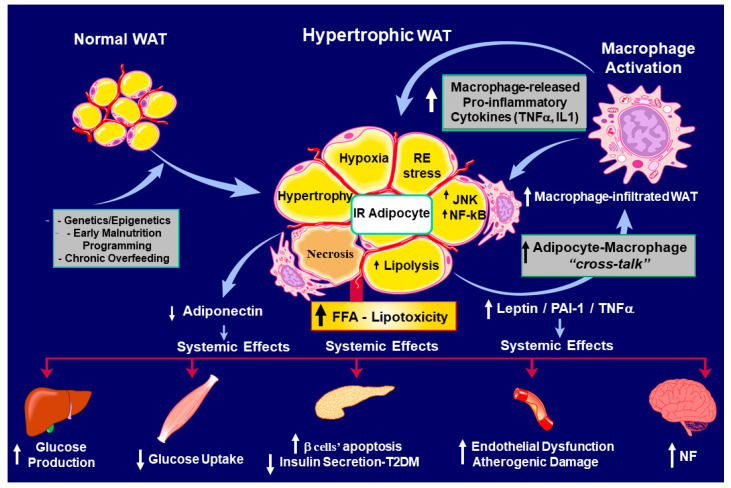
Inflamed white adipose tissue (WAT) is characterized by large insulin-resistant (IR) adipocytes. WAT mass is now highly infiltrated by macrophages; pro-inflammatory adipocytokines and free fatty acids (FFAs) will be released in excess. Thus, a chronic overall IR state induces several dysfunctional tissues: enhanced hepatic glucose (GLU) production, hyperglycemia, diminished muscle glucose uptake, pancreatic β-cell apoptosis, and type 2 diabetes mellitus, and consequently, neuroinflammation (NF) is established (adapted from Pagano et al. [42]).

These hypertrophic cells are seriously compromised in IR and highly secrete pro-inflammatory adipocytokines. In turn, hypertrophic WAT cells are recognized as altered cells, and consequently, the tissue is infiltrated by cells from the immune system, mainly macrophages, which secrete pro-inflammatory cytokines, mainly TNF-α, IL-1, and IL-6. This inflammatory loop generates a vicious cycle that may chronically perpetuate and promote an inflammatory disorder. In this scenario, the individual is at a high risk of an established dysmetabolic state, accepted as metainflammation [41], resulting in the appearance of co-morbidities, thus compromising the individual’s health [42], and if this condition is not rapidly counteracted, neuroinflammation will follow until it is resolved (Figure 2).

**Figure 3:**
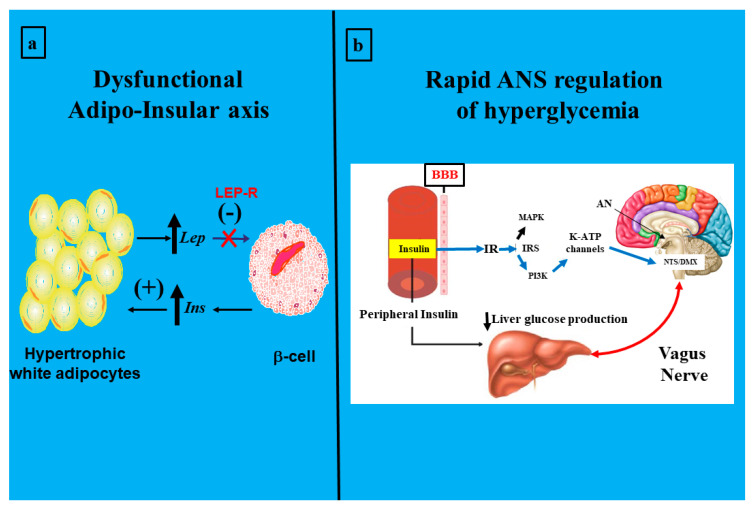
(**a**) A disrupted adipo-insular axis function during leptin-resistance (LEP-R). (**b**) The rapid, vagus-mediated regulation of hyperglycemia shortly after triggering the insulin-resistant (IR) state Lep, leptin; Ins, insulin; *β-cell*, pancreatic β cell; BBB, brain–blood barrier; IRS, insulin-receptor substrate; MAPK, mitogen-activated protein kinase; PI3K, phosphatidylinositol 3-kinase; K-ATP, potassium-dependent ATP; AN, arcuate nucleus; NTS, nucleus tractus solitarius; DMX, dorsal motor nucleus X (panel b was adapted from Prodi and Obici [49]).

The obesity epidemic and T2DM, originally called the *twin* epidemic, actually combines with a critical component: IR; this is now known as the *triplet* epidemic. This is not a minor concept, because the astonishing growth of these problems seriously affects global public health due to the difficulties of the early diagnosis of the IR state. Metabolic abnormalities during a chronic insulin-resistant state of an organism end in cardiovascular-disease-related death. They could therefore be called *silent killers*. Research has been essential in developing the idea of an association between obesity and diabetes. Thus, it has progressively been linked not only with obesity’s severity but also to weight gain and duration of weight catch-up. Numerous experimental works and epidemiological and clinical studies have cemented an interesting story that, in part, shows the *stormy* (excessive release of pro-inflammatory cytokines) relationship between obesity and T2DM [43,44]. Indeed, changes in tissue and peripheral lipids could be considered the most prominent promoters of IR [45,46,47]. It is imperative to recognize that WAT is essential for regulating energy expenditure and, therefore, for life. However, the problem appears when this functionality is broken and the adipocyte is no longer a *friendly* component but a *foe*. Hyperglycemia takes place, among others, through a disrupted adipo-insular axis function (Figure 3, panel a). Physiologically, a stimulatory effect of insulin on adipocyte leptin production (synthesis and release) occurs; reciprocally, direct leptin-inhibitory activity on β-cell insulin production occurs [48]. Thus, when this endocrine axis becomes dysfunctional, compensatory activity through the ANS (vagus nerve) [49] could be triggered to rapidly modulate glycemia (Figure 3, panel b). Leptin circulating levels directly correlate with body fat mass and increase after food intake, whereas they decrease during fasting [50,51]. Physiologically, insulin acts directly on adipocytes, increasing *ob* expression and secretion [52]. In turn, leptin reduces pancreatic β-cell function, thus decreasing insulinemia. Leptin’s in vivo role in glucose metabolism has been proposed to be mediated by the ANS [53]. 

White adipocyte multiple adipokine production (e.g., TNF-α, IL-1, and IL-6) [54,55] favors the increase in leptin WAT and other pro-inflammatory adipokines [56]. Leptin is key in controlling food intake and in regulating energy expenditure by inducing satiety through hypothalamic Ob-Rb activity [57,58], a mechanism mediated through the JANUS/STAT pathway [59]. Leptin inhibits lipogenesis and increases β-oxidation, adipocyte apoptosis, and UCP-1 expression through both autocrine mechanisms and ANS efferent pathways [60]. Thus, as mentioned above, leptin inhibits insulin and thereby affects glucose homeostasis by combining with its hypothalamic effect [61]. Conversely, leptin inhibits glucagon production in pancreatic α-cells [62]. Therefore, the *adipose–insular* axis plays a predominant regulatory role in energy balance, wherein insulin and leptin are the active peripheral messengers. Consequently, dysfunction of this axis facilitates both obesity and T2DM development [63]. LEP-R, mainly at the hypothalamic level, is a characteristic in most obese phenotypes [59,64,65]. Energy homeostasis requires a precise balance between metabolic substrate utilization by the brain and peripheral organs and by exogenous (diet) or endogenous (liver, gut, adipose tissue, and kidney) arrangement. The main physiological objective of glycemia is to guarantee an appropriate supply of GLU to the brain and other tissues, including the gut [66]. Therefore, dysfunction of the adipose–insular axis may facilitate obesity-associated metainflammation (e.g., dyslipidemia, metabolic syndrome, T2DM) [63].

### 3.3. Hypothalamic-Mediated Mechanisms Involved in Neuroinflammation

Leptin resistance is a characteristic in some obese phenotypes, and several hypothalamic mechanisms have been proposed to explain this process [59]. As mentioned above, energy homeostasis requires a precise balance between metabolic substrate utilization (mainly GLU and FFAs) by the brain and peripheral organs and exogenous (diet) or endogenous tissue arrangement. The main physiological objective is a GLU peripheral level that guarantees an appropriate GLU supply to the brain and other organs. Once metainflammation is triggered, it must be considered that the hypothalamus plays a key role in whole-body energy homeostasis and metabolism [65]. This communication is due to the arrival of peripheral signals (e.g., hormones, adipo-/cytokines, metabolites) translating their activities into the CNS. Signals received by different hypothalamic nuclei (namely, the ARC nucleus) induce many peripheral functions susceptible of being modified once neuroinflammation is triggered. Various reports indicate that several obese phenotypes, including that from high-fat diet (HFD) intake, modify CNS energy homeostasis [66,67,68,69]. In fact, an HFD triggers hypothalamic neuroinflammation after activating the toll-like receptor 4 (TLR4) [70]. Moreover, many undesirable cellular mechanisms merge, such as oxidative stress (OS), namely, at the endoplasmic reticulum (ER) level, and the up-regulation of the SOCS3 and IKKβ/NF-κB pathways [71]. HFD-induced obesity also activates immune cells inside the CNS (microglia and astrocytes), thereby worsening neuroinflammation [71,72,73,74]. It has been demonstrated that HFD-induced hypothalamic neuroinflammation occurs earlier than developing peripheral inflammation and hypertrophic expansion of WAT mass [66,72]. Furthermore, the inhibition of microglia functionality and hypothalamic blocking of neuroinflammation-mediated pathways could prevent obesity-dependent dysmetabolism [66,72,73,74] to cope with neuroinflammation. Diet-induced obesity (DIO) activates the inflammatory process at the hypothalamic level; as a consequence, enhanced production of pro-inflammatory cytokines and impairment in both insulin and leptin signaling pathway mechanisms develop in an overall insulin- and leptin-resistant individual phenotype of neuroinflammation [75].

As previously mentioned, the BBB is an essential structure that protects the brain against peripheral inflammation. Therefore, a change in its function is relevant for developing and maintaining neuroinflammation. Modifying BBB permeability [11,76] can result in an increased translocation of circulating inflammatory signals, such as soluble factors, cells, or pathogens, resulting in one of the main causes of neuroinflammation and enhanced susceptibility to ND development. However, other brain structures are affected and are responsible for neuroinflammation in combination with the injured hypothalamus. Pertaining to the limbic system, the hippocampus is a structure located in the inner area of the temporal lobe and is particularly important in regulating emotional responses. Indeed, hippocampal impairment can be found in the early phases of NDs. This structure is highly susceptible to damage by obesogenic factors, mainly saturated FFAs [76,77], which, in turn, contribute to hippocampal neuroinflammation and thereby to ND development [72,73,74]. Obesogenic diets can enhance the expression of pro-inflammatory cytokines in the hippocampus and, in turn, activate the microglia [75]. As a result, hippocampal neuroinflammation alters neuronal communication and metabolism once triggered, which is similar to what occurs in hypothalamic neuroinflammation [66,76,78], and notices the modification of BBB permeability [76]. The main cellular structure involved in regulating energy homeostasis is the mitochondria. In obese individuals, mitochondrial dysfunction (decreased energy metabolism) due to dysfunctional glucose transport is a key altered mechanism for retaining fat energy [79], cooperating greatly with brain dysfunction and IR [80,81,82,83,84].

Moreover, mild enhanced chronic glucocorticoids in the periphery (from both hypertrophic adipocyte metabolism and established corticoadrenal leptin-resistance) could inactivate the counter-regulation of white to brown-like adipocyte trans-differentiation (*browning*) due to a glucocorticoid-receptor-mediated mechanism [85], thereby cooperating with the decreased dissipation of body energy. Indeed, ROS generation exceeds antioxidant activity, causing cellular oxidative damage. WAT OS is a major contributor to IR and cellular dysfunction [86] and is regulated in a depot-specific manner. Also, WAT OS varies with age; compared to younger mice, aged C57BL/6 mice exhibit increased ROS in visceral WAT (namely, epididymal and epicardial WATs) [87,88]. Oxidized lipids and proteins accumulate in the visceral fat pad [89,90]. Consequently, significant challenges exist in defining the specifics of ROS-driven pathology and its connection with human obesity and T2DM (see Figure 2).

### 3.4. Insulin-Resistance-Related Neuroinflammation in Neurodegenerative Diseases

**Figure 4:**
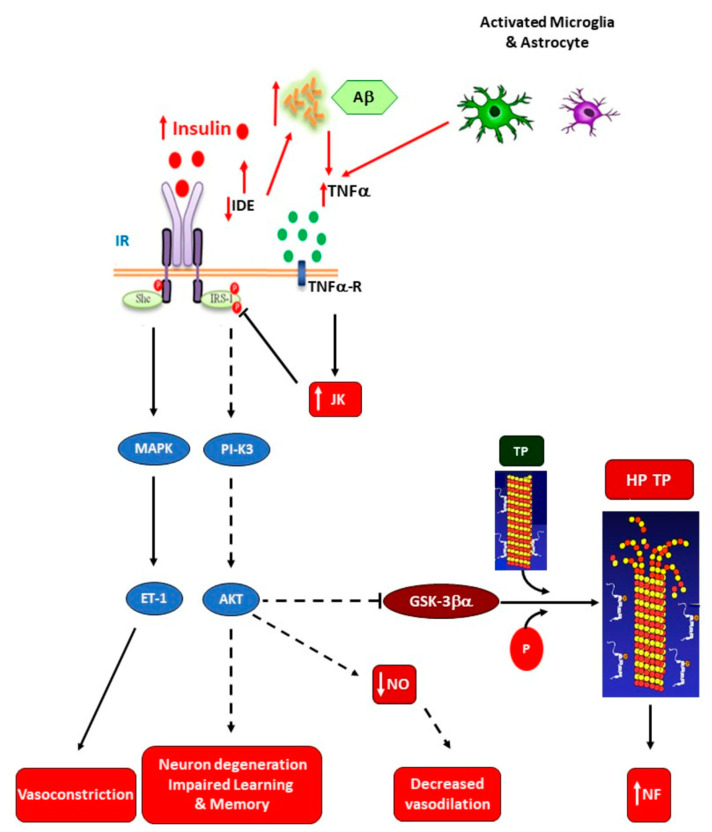
Disrupted brain insulin signaling in Alzheimer’s disease. Amyloid-β (Aβ) accumulation increases TNF-α levels and activates c-Jun N-terminal kinase (JNK). Consequently, IRS-1 becomes inhibited. IR decreases insulin-degrading enzyme (IDE) expression, thereby diminishing IDE-induced Aβ degradation. Reduced brain insulin signaling decreases the inhibition of glycogen synthase kinase-3β (GSK-3β) activity on tau protein (TP) phosphorylation, resulting in hyperphosphorylated TP (PP-TP) production and microtubule depolymerization (MTDP). Consequently, neurofilament tangles (NFTs) deposition is augmented. Therefore, impaired insulin signaling induces neuron degeneration and modifies learning and memory (AD). Additionally, IRS-1 deficiency reduces nitric oxide (NO) production and up-regulates endothelin-1 production, thereby decreasing brain blood flow and increasing neuroinflammation (NF) (reproduced with Karger AG’s permission from Spinedi and Cardinali [20]).

Brain neuroinflammation may be extremely dangerous for the development of NDs. Indeed, once the overall IR state is established, a high risk for the development of AD by a mechanism mainly dependent upon poor brain insulin signaling could be established. This is supported by the fact that brain IR can promote local tau pathology and amyloidogenesis [91]. Moreover, an increased risk of developing T2DM has been reported in AD patients [92], which supports the interrelationship between brain lesions and metabolic disturbances in AD patients, including the deterioration of cognitive function [93]. Functional insulin signaling has been reported to promote neuron plasticity and ameliorate memory in humans treated with intranasal insulin [94]. The IR developed in the AD brain appears to be related to Aβ plaques and tau pathologies [95], as supported by inhibited brain IRS-1 in patients showing tauopathies [96], wherein Aβ oligomers promote insulin receptor internalization [97]. Also, an enhanced activation of c-Jun N-terminal kinase, protein kinase R, and TNF-α takes place and, in turn, inhibits IRS-1 function [95,98]. All these observations strongly support the fact that impaired glucose homeostasis in AD patients seems to be closely related to brain (hypothalamic) damage due to local IR (abnormal insulin signaling), thus augmenting brain TNF-α production and probably perpetuating neuroinflammation (Figure 4).

**Figure 5:**
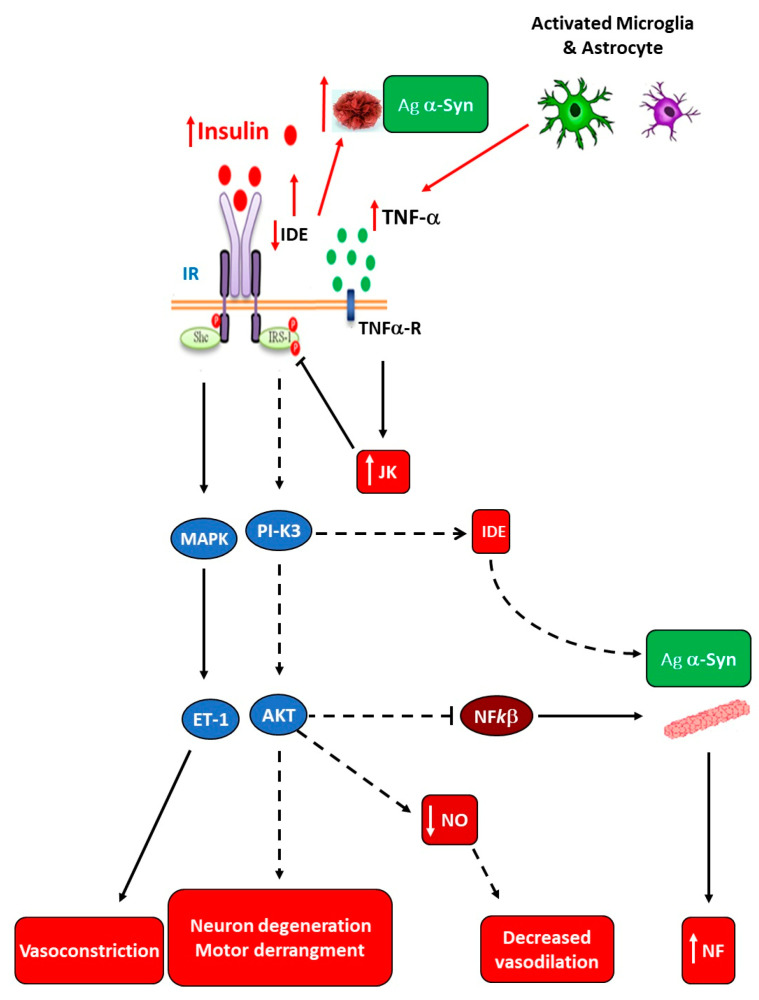
Disrupted brain insulin signaling in Parkinsos’s disease. Amyloid-β (Aβ) accumulation increases TNF-α levels and activates c-Jun N-terminal kinase (JNK). Consequently, IRS-1 becomes inhibited. IR decreases insulin-degrading enzyme (IDE) expression, thereby diminishing IDE-induced Aβ degradation. Diminished brain insulin signaling decreases inhibition of NF-*k*B activity on α-Synuclein (α-Syn) aggregation (panel b), thereby accumulating it. Consequently, impaired insulin signaling induces neuron degeneration and modifies motor activity. IRS-1 deficiency also reduces nitric oxide (NO) production and up-regulates endothelin-1 production, thereby decreasing brain blood flow and increasing neuroinflammation (NF) (adapted with Karger AG’s permission from Spinedi and Cardinali [20]).

The pathophysiological causes of Parkinson’s disease (PD) are still unclear; however, IR, mitochondrial dysfunction, OS, and neuroinflammation seem to be the most critical disease-related dys-mechanisms [99,100,101]. Unfortunately, at present, dopaminergic neuron death and PD progression appear to be indomitable events only susceptible to palliative treatment for motor dysfunction through drug therapy, such as dopamine (DA) precursor (DOPA) and DA agonists. As shown in Figure 2, hypertrophic adipocytes highly secrete TNF-α and increase NF*k*B expression, thereby augmenting their signaling. In the IR-established endogenous environment during the hypertrophic expansion of WAT mass, the PI3K/AKT signaling pathway becomes disrupted. Then, a reduced or a lack of inhibition of NF*k*B production and the modification of other cell function mediators (e.g., GSk3β, FOX01, mTOR) occur. In turn, mitochondrial dysfunction and α-Synuclein (α-Syn) aggregation are the leading causes of death of dopaminergic neurons [102], resulting in neuroinflammation. Moreover, pancreas-secreted islet amyloid polypeptide (IAPP) can cooperate with α-Syn aggregation, so that reduced neuroprotection aggravates PD progression [103] (Figure 5).

In summary, it is thus important to monitor the detrimental health consequences derived from inflamed, insulin-resistant WAT. This condition, if not appropriately controlled, will result not only in metainflammation but also in chronic hyperglucemia (T2DM), a hypertrophic obesity phenotype, and an increased risk of cardiovascular events. Moreover, a circulating excess of pro-inflammatory factors will induce a dangerous neuroinflammatory condition and, thus, neurological disorders.

## 4. Gut and Neuroinflammation

### 4.1. Role of Microbiota in Neurodegenerative Diseases

**Figure 6:**
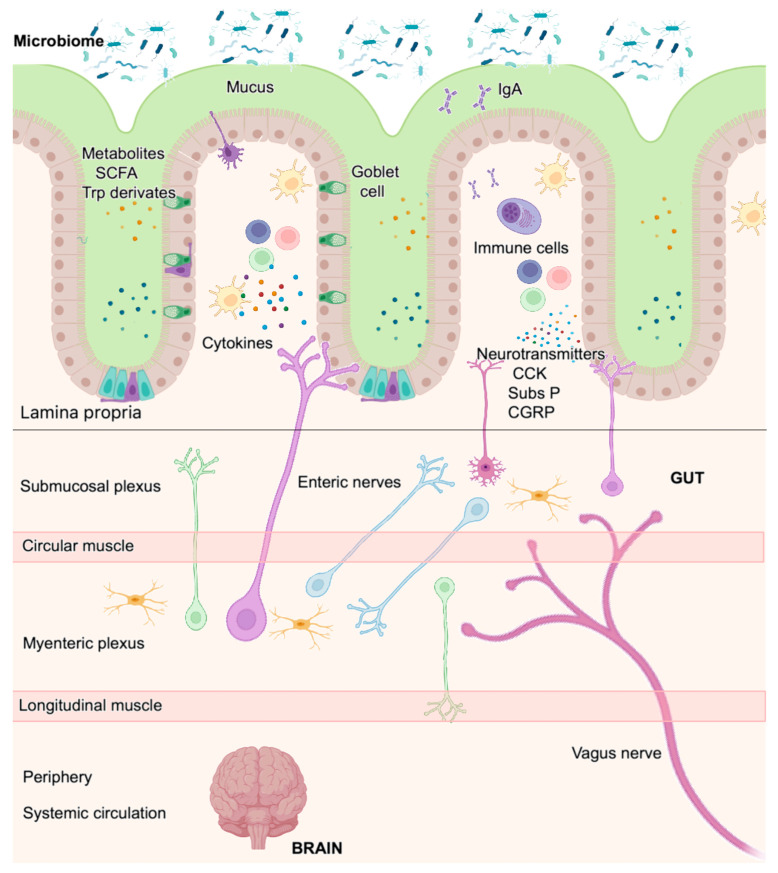
Schematic representation of the gut–brain axis (GBA), including components of the intestinal microbiome, which interacts with nervous and immune cells. TNF-alpha, tumor necrosis factor-alpha; IL-1β, interleukin-1β; IL-6, interleukin 6; GLP-1, glucagon-like peptide-1; PYY, pancreatic polypeptide peptide tyrosine-tyrosine; substance P, Subs P; CCK, cholecystokinin; SCFA, short-chain fatty acid.

Supporting evidence highlights the importance of the microbiota–gut–brain axis function in NDs [104,105,106] (Figure 6). Indeed, an interaction between gut microbiota and microglia in AD has been found. In homeostatic conditions, the gut microbiome regulates microglial maturation and activation via short-chain fatty acid (SCFA) release [105]. In the above study, the authors found that germ-free and antibiotic-treated mice suffered from impaired microglial immune responses when challenged with bacterial lipopolysaccharide and lymphocytic choriomeningitis virus infection. However, microglial defects were partially restored by recolonization with complex microbiota and SCFA supplementation [105]. Similarly, regarding PD, the gut microbiota appears to be a key factor in this ND. Gastro-intestinal symptoms and altered gut microbiota are found in PD patients [107]. Several pieces of evidence [108,109] demonstrated that the gut microbiome was influenced by development of α-Syn pathology, microglial activation, and motor deficits in α-Syn-overexpressing (ASO) mice that appeared to be influenced by the gut microbiome. This became evident in experimentation with SPF ASO mice, which exhibited greater PD pathological signs than their germ-free and antibiotic-treated counterparts. Remarkably, the treatment of germ-free ASO mice with fecal microbes from PD patients restored the primary disease, i.e., α-Syn-mediated motor dysfunctionality [108]. Altogether, studies support the relevance of the microbiota–gut–brain axis in the pathogenesis of ND, such as AD and PD [109].

### 4.2. The Gut–Brain Axis: Role of the Hypothalamus

The presence of a pathogen within the gut mucosa triggers the induction of mechanisms that may lead to excessive inflammation and permanent tissue damage with disturbance of tissue homeostasis. The gut–brain axis (GBA) (Figure 6) is a bidirectional communication network linking the ENS and CNS with intestinal cells, allowing the brain to influence intestinal functions, from gut organogenesis to effector immune mechanisms, and the gut to influence mood, cognition, and mental health [110,111,112]. This complex GBA integrates emotional and cognitive brain centers with the intestinal function to fine-tune the immune response to invading pathogens and tissue damage, restoring homeostasis.

Indeed, once external threats by pathogen invasion and other injuries are detected, neurons can directly sense them and rapidly communicate with immune cells and vice versa. Soluble factors are essential in this crosstalk, while neurons can interact with many cells [macrophages, neutrophils, dendritic cells (DCs), epithelial cells, mast cells, innate lymphoid cells (ILCs), and adaptive cells (B and T lymphocytes)] to promote the antimicrobial response [113,114].

The ANS, the hypothalamo-pituitary–adrenal (HPA) axis, and nerves in the gastrointestinal tract link the gut and brain directly and indirectly. Their behavior and immunity are synchronized during infection to halt the spread of microorganisms (e.g., fever, lethargy, anorexia, social isolation), and this has been thoroughly described. Secreted pro-inflammatory cytokines from innate cells affect the HPA axis function. Then, produced cortisol impacts the liver, promoting acute-phase protein production (amyloid A protein, fibrinogen, C-RP, complement components, mannose-binding protein, coagulation proteins, etc.), thus exerting immune functions. A gut–vascular barrier that controls the passage of different components and microorganisms from the intestinal lumen to the blood, preventing the dissemination of bacteria to the liver and other tissues, was recently described [13,14]. The GVB and the BBB are interconnected within the GBA, which is critical to understanding how inflammation and psychosocial disruptions can affect homeostasis.

### 4.3. The Nervous System and Gut: Evidence of Cell Interactions

As mentioned above, the intestine is continuously exposed to microorganisms and derived metabolites. Hence, the gastro-intestinal tract is densely innervated and contains most of the organism’s immune cells. This environmental antigenic challenge makes the intestinal mucosa the main body sensory interface. Indeed, immune cells sense microorganisms, their metabolites, and stress and signals from damaged tissues through different pattern-recognition receptors. Exogenous pathogen-associated molecular patterns (PAMPs) (e.g., flagellin, LPS CpG, etc.), endogenous membrane-bound secreted stress molecules, and damage-associated molecular patterns (DAMPs) (e.g., decreased intracellular K^+^, extracellular ATP, uric crystals, sustained hyperglycemia, etc.) activate cells bearing PRRs. Then, the activated cells communicate with neighboring cells (immune cells and neurons, or innervating fibers) to promote inflammation that modifies gut motility, immune cell recruitment and activation, and gut permeability, thereby interfering with CNS function (energy homeostasis, food intake, etc.). Sensitive and enteric neurons can also directly detect microorganisms at barrier tissues by expressing different PRRs, such as TLR7 or TLR2/TLR4, and, in turn, secrete neurotransmitters and neuropeptides. Understanding neuro-immune interactions and their impact on barrier tissue physiology has led to the development of novel tools to characterize neurons and immune cells [114].

ANS components have been described to control the main inductive sites of the gut from embryonic life to control lymphocyte activation and differentiation [115,116,117,118]. The ENS is involved in developing Peyer’s patches, vital inductive tissues for generating mucosal immunity in the gut [117]. Moreover, sympathetic nerves located close to Peyer’s patches also innervate the submucosa and muscularis and control blood flow and cell distribution. Although Peyer’s patches are distributed along the human small intestine, the highest density of them is found at the ileum, which is highly innervated compared to other intestinal mucosa. This suggests the role of fibers and neurons in immune cell activation. Cells located in the follicle dome and B and T areas (macrophages, dendritic cells, B cells, T cells, and IgA-producing plasma cells) are proximal to projections from the submucosa plexus, a site that includes intrinsic sensory and motor neurons as well as extrinsic sympathetic neurons [118]. These cells also express type 2 muscarinic acetylcholine receptors to detect neurotransmitters [119]. This means that noradrenergic fibers modulate cytokine and chemokine secretion from B and T cells and also control immunoglobulins released by plasma cells through binding to adrenergic receptors [120,121]. At the muscularis site, neuron–macrophage interactions control TNF-α secretion and phagocytosis [122], and parasympathetic innervation of the gut, mainly through the vagus nerve, also controls the ENS, barrier function, inflammation, and immunity [123]. Intestinal macrophages, a heterogeneous cell population, possess differential functions depending on tissue location and are also highly concentrated and distributed in different intestinal wall layers. Lamina propria macrophages (LpMs) are located under the mucosa’s epithelial layer and close to gut lumen. Macrophages and dendritic cells are sentinel cells that surveil luminal microorganisms. They adapt to environmental changes, respond to microorganisms that can translocate within intestinal tissue, and promote tolerogenic circuits for harmless antigens, such as food [124]. LpMs extend protrusions through the epithelium to capture bacteria directly from the intestinal lumen through the tight junctions [125] without disrupting barrier integrity. The chemokine fractalkine (CX3CL1), secreted by epithelial cells, controls this mechanism by interacting with the fractalkine receptor (CX3CR1) expressed in LpMs [126,127]. Then, the interaction of the intestinal lamina propria resident CX3CR1^+^ LpMs with migrating CD103^+^ dendritic cells promotes the homing of CCR7^+^ CD103^+^ dendritic cells with antigen cargo to mesenteric lymph nodes [128,129]. This mechanism is critical for inducing specific tolerogenic circuits to harmless antigens [130,131].

Nevertheless, macrophages can be detected distant from the lumen, in the outer smooth muscle layer. Muscularis macrophages (MMs) have a stellate morphology, are positioned along nerve fibers adjacent to neuron bodies, and are endowed with a tissue-protective phenotype to counteract inflammation [132]. Interestingly, LpMs are sensitive to luminal changes, whereas MMs are sensitive to tissue changes. The environment’s perturbation triggers a differential activation gene profile in gut-resident macrophage subsets and promotes specialized functions. The expression of a higher level of membrane β2 adrenergic receptors in MMs than in LpMs may reflect the proximity of muscularis macrophages to the neuronal network. Gut extrinsic SNS innervation of the muscularis layer has amply been shown to primarily contribute to the catecholaminergic response through NE secretion. MMs respond by inducing *Arginase 1* (Arg1) expression, while *Tnf*-gene expression remains unchanged upon intestinal infection [133]. Arg1 is implicated in tolerogenic immune mechanisms and preserving neurons from apoptosis [134]. The neuron-immune unit counteracts intestinal over-inflammation to avoid tissue damage with neuron loss. In a steady state, MMs are also involved in critical intestinal functions, such as the modulation of gastro-intestinal motility due to the secretion of bone morphogenetic protein 2 (BMP2), a factor that impacts enteric neurons expressing BMPR. Likewise, neurons respond by secreting the macrophage colony-stimulatory factor (M-CSF). Commensal bacteria promote this bidirectional interaction, as germ-free [135] or antibiotic-treated individuals [128] exhibited severe dysmotility with a lower expression of *Bmp2* and *Csf1 and* lower frequency of MMs, together with no changes in the number of LpMs, compared with normal individuals [136].

The interaction between ENS, SNS, and PNS neurons innervating the gut muscularis and lamina propria with tissue-resident MMs and LpMs, respectively, is involved in the neuro-immune unit’s adaptation to different tissue niches in response to environmental perturbations and in maintaining resistance and tolerance balance to cope with allostasis [136].

The neuro-immune unit also contains mast cells proximal to nerve fibers, mainly in the mucosa and submucosa [137]. Enteric neurons secrete the growth factor Kit ligand, a factor interacting with the tyrosine-kinase receptor Kit on mast cells, which is essential for cell maintenance [138,139]. Nociceptor neurons are sensory neurons containing several cytosolic neuropeptides (e.g., substance P, vasoactive intestinal peptide, calcitonin-gene-related peptide, or CGRP) that can be released during inflammation, modulating immune and stromal cells. These peptides activate vascular smooth muscle, endothelial, epithelial, and mast cells. In particular, substance P triggers mast cell degranulation accompanied by visceral pain, diarrhea, and dysmotility. This crosstalk between neurons and mast cells is essential in maintaining a steady state for gut homeostasis and in inflammatory conditions (such as parasite infection, irritable bowel syndrome, and food allergy). These circumstances can be accompanied by gastro-intestinal motility disturbance and promoted by mast cell degranulation at a high frequency [140,141,142].

Nociceptor sensory neurons also control epithelial barrier integrity and homeostasis through CGRP, substance P, and mucus secretion. Goblet cells are specialized epithelial cells that produce mucins and enzymes that form the outer mucus layer to prevent the contact of microbes with the apical face of epithelial cells and microbial penetration [143]. Commensal and food antigens have been described to promote neuronal CGRP secretion, which interacts with Ramp1 on goblet cells to drive mucus secretion. Deficiencies in the CGRP–Ramp1 axis show decreased mucin production and susceptibility to colitis [144].

Enteric neurons also coordinate the function of innate lymphoid cells (ILCs) in the gut during early immunity. ILCs lack PRR but produce vast amounts of cytokines in response to tissue disturbances [145]. Tissue-resident ILCs react to alarmins and cytokines, hormones, prostaglandins, and metabolites (e.g., aryl hydrocarbon receptor ligands, vitamin A) and are sensitive to neurotransmitters and neuropeptides [146]. The different subsets of ILCs are located close to SNS, PNS and ENS neurons, and crosstalk between cells regulates activation or suppression depending on the context [147]. Vagus nerve acetylcholine (ACh) acts as an anti-inflammatory factor that suppresses pro-inflammatory type 2 cytokine release from ILC2 cells via the α7-nicotinic Ach receptor and is involved in infection resolution, allergy, and autoimmunity [148,149]. The anti-inflammatory and host-protective roles of ACh were also described in macrophages and ILC3 cells for coping with infections [150]. Norepinephrine (NE), an SNS-derived neurotransmitter, also inhibits ILC2 by binding to β2-adrenergic receptors. The role of NE was described in activation, proliferation, and type 2 cytokine secretion in helminth infections and allergic gut and lung inflammation [151]. Moreover, neuromedin U, a neuropeptide derived from ENS neurons, rapidly and robustly stimulates ILC2 through neuromedin U receptors for type 2 pro-inflammatory cytokine secretion during gut and lung worm infection [146,151,152]. ILC1 cells are also sensitive to the suppressive effects of glucocorticoids (GCs) through GC-Rs; LPS-induced IFN-γ secretion can be limited in mice by a direct GC effect on NKp46^+^ ILC1 cells [153]. GCs also inhibit ILC2 and CD4^+^T cells in lung allergic inflammation [154]. Finally, the food-induced enteric neuron-derived vasoactive intestinal peptide (VIP) also stimulates ILC2 cells through VIP receptor 2 to trigger type 2 inflammation [155]. VIP also stimulates IL-22 secretion by ILC3 cells, and this cytokine promotes barrier integrity and the production of antimicrobial peptides by intestinal epithelial cells [156]. VIP production after food intake indicates that enteric neurons prepare the intestinal mucosa for tissue preservation. In summary, ILCs are primary sensors of neuronal factors, and crosstalk between ILCs and neurons is essential for tissue homeostasis regulation.

Remarkably, neurons also modulate the adaptive immune response. The expression of Coh-/NE-gic or neuropeptide receptors on dendritic cells, which act as a nexus between innate and adaptive immunity, suggests that neurons regulate DC function. Stimulation of β-2 adrenergic receptors abrogates cross-presentation with further Th1 suppression and Th2/Th17 induction [157]. Conversely, α-adrenergic receptor stimulation enhances antigen uptake and migration for T cell activation [158]. VIP showed a regulatory function through Treg differentiation [159], and catecholamines have been reported to impact B and T cell functions, promoting lymphocyte recruitment, Th2, Th17, Treg, and antibody responses [160,161].

These data demonstrate that neuro-immune crosstalk is coordinated to detect the presence of microorganisms or tissue injury, exerting an intricate circuit of activation and modulation to restore tissue homeostasis. This is critical in tissues where cells, such as neurons, have no or reduced migratory properties and proliferative potential to regenerate and re-populate. Mucosal neurons and immune cells have adopted common sensing strategies and are equipped with specific PRRs to detect microbes directly. After immune cell activation, effector protective mechanisms are induced, and an anti-inflammatory response is promoted to preserve intestine homeostasis. Immune cells and neurons can develop different mechanisms to solve inflammation and tissue repair during restoration of homeostasis. Uncontrolled and chronic exaggerated inflammation drives many intestinal diseases, e.g., food allergy, intestinal BD, irritable bowel syndrome, and infectious disease with septic shock [113]. Animal models and human clinical trials have demonstrated that vagus nerve stimulation controls chronic inflammation and alleviates pathological conditions such as Crohn’s disease and rheumatoid arthritis (RA) in humans [162,163] and colitis, hypovolemic shock, endotoxemia, and RA in experimental models [163,164,165].

### 4.4. Role of the Microbiota in Tissue Barriers

The gut–brain axis was originally described as a unidirectional signaling network that regulated intestinal functions from the CNS in a top-down manner. Nevertheless, extensive studies on the intestinal microbiota have described complex interaction between microbes and the mucosal immune system in the 21st century and have revealed the bidirectional regulation of neurons and immune cells. In the last decade, intestinal microbes were included in the GBA, and we are starting to understand how the enteric microbiota influences the gut–brain relationship (e.g., mental state, emotional regulation, neuromuscular function, regulation of the HPA axis, development of mucosal immune structures, regulation of mucosal immune cells, energy homeostasis, etc.). Commensal microorganisms or their metabolites modulate the CNS and PNS, and sensing microbes is not always considered an injurious signal. The gut microbiota communicates with the CNS through the vagus nerve and systemic circulation and also through the local nervous and immune systems (Figure 6). Nervous and immune cells can sense microorganisms and their metabolites and promote inflammation when dysbiosis is present. Conversely, immune and nervous cells can act on the microbiota composition to restore microbial homeostasis. Gut-innervating TRPV1^+^ sensory neurons or nociceptor cells produce substance P and CGRP that promote tissue protection and barrier integrity. Chemoablation of these cells promotes intestinal damage, inflammation, and dysbiosis. Colonization of germ-free mice with a consortium of Gram^+^ *Clostridium spp* or the administration of substance P restored microbial homeostasis and limited the severe gut inflammation of nociceptor-defective mice.

Intestinal barrier integrity is critical to promoting this bidirectional control and the translocation of metabolites. Different vascular and epithelial barriers, such as the intestinal epithelial barrier, gut–vascular barrier, blood–brain barrier, choroid plexus vascular barrier, and blood–cerebrospinal fluid barrier, are also critical to maintaining a regulated network for local and systemic functions [166]. Maintaining the epithelial barrier and vascular structures in the gut promotes the correct exchange of metabolites between the lumen and the mucosal tissue to modulate selective permeability. The microbiota is implicated in preserving a functional GVB and, strikingly, the integrity and permeability of the BBB. Germ-free mice exhibit a highly leaky BBB with impaired expression of tight-junction proteins, resulting in distal neurological disease [167]. During dysbiosis, the GVB is disrupted, gut permeability is increased, bacteria are translocated, and metabolites enter the circulation, causing local and distant inflammation [166]. It was documented that antibiotic-induced dysbiosis led to GVB dysfunction and liver disease [168], and GVB disruption in a colitis model provoked a leaky cerebral vascular barrier [14]. It can be assumed that the GVB–BBB axis represents a multifaceted additional alliance that connects the gut with the brain and that the microbiota is critically involved in controlling its integrity and local and distal inflammation. Microbial dysbiosis, which has been implicated in inflammatory bowel diseases and irritable bowel syndrome, may be considered a comorbid condition for neuroinflammatory processes such as AD, PD, and/or depression [169,170,171].

### 4.5. Crosstalk Between the Intestinal Microbiome and Nervous System

Enteric and extrinsic neurons can directly sense the microbiota, and the microbiota can modulate neuronal functions by producing short-chain fatty acids (SCFAs) such as propionate, acetate, butyrate, etc., [172]. An exaggerated HPA stress response described in germ-free models was partially mitigated during early exposure to microbial commensals [173]. This work revealed a link between microorganisms, cognitive processes, and neurological and psychological functions [172,174,175,176]. Also, microbial dysbiosis has been linked to the pathology of prevalent neurological conditions, including autism, depression, AD, and PD [177,178]. Different gut microbial metabolites have been described to traffic to the brain through circulation. The vagus nerve is deeply involved in this bidirectional interaction, linking the luminal bacteria content with the brain. Microbes and microbial components trigger vagal responses regardless of whether they are pathogenic or commensal [179]. Early *Citrobacter rodentium* infection increased vagal activation and anxiety-like behavior in rodents [180], whereas exposure to commensal *Lactobacillus johnsonii* enhanced vagal nerve activity, lowering blood pressure [181]. LPS is also known to elicit broad neuroinflammatory effects, including alterations at the site of the CNS–blood interface, the BBB, following injection into the bloodstream or cerebral spinal fluid [182,183]. Early exposure to intestinal microorganisms also impacts BBB formation and integrity. Germ-free mice exhibit impaired BBB development and increased permeability. Microbial colonization or exposure to microbial-produced SCFAs increases the expression of endothelial tight-junction proteins, significantly improving BBB integrity [167]. In conventional mice, antibiotic treatment decreases microbial diversity and intestinal SCFA levels. Antibiotic treatment also alters the expression of tight-junction proteins in the hippocampus and amygdala, further suggesting a link between SCFAs and BBB integrity [176]. Propionate was recently reported to protect cultured BBB endothelial cells from inflammatory and oxidative stresses via the receptor FFAR3 [183,184,185,186,187,188,189]. The diet is a critical point in maintaining the microbiome and local and systemic homeostasis.

### 4.6. The Role of Diet in Microbiota Composition and Neuroinflammation

There is accumulating evidence in rodent models showing that high-fat diet consumption leads to dysbiosis with enhanced susceptibility to inflammation and hyperphagia with obesity [190,191]. It has been demonstrated in rats that chronic high-fat diet consumption induces vagal inflammation, which impacts the brainstem, dysbiosis, and overfeeding with weight gain. HFD consumption leads to vagal hypofunction, inflammation, and regulation of food intake [192]. Furthermore, HFD-driven inflammation has been linked with a dysbiotic microbiota [193]. The colonization of microbiota-depleted rats with microbiota from rats fed with a conventional diet induced vagal remodeling, controlled inflammation, and normalized food intake [194].

As described above, different cells can synthesize and secrete neurotransmitters and neuropeptides, and specific bacteria from the microbiota can generate or metabolize precursors from the diet, potentially limiting their availability to the host. Tryptophan (Trp) is an essential ingested amino acid, and microbes in the intestinal lumen metabolize it. Germ-free mice showed elevated levels of serum Trp and its metabolites kynurenine and serotonin, which were reduced following colonization [195]. Patients with IBD suffering from depression [184,196] showed low levels of Trp in serum [185]. Mice deprived of Trp also showed enhanced susceptibility to colitis [186], which was reverted with Trp administration [187].

Furthermore, serotonin is a crucial neurotransmitter involved in modulating intestinal peristalsis, innate and adaptive cell functions, anxiety, and depression. Depression-like behavior induced in rats was reverted with serotonin diet supplementation [197]. This means that the microbiota is implicated in Trp availability and serotonin synthesis, and the balance between utilization and metabolization impacts Trp and serotonin availability. In conclusion, alterations in microbiota composition may lead to gut inflammation, depression, social–cognitive deficits, visceral pain, dysmotility, and others. Administration of probiotic *Byfidobacterium* capable of producing Trp metabolites or supplementation with Trp or food-derived bioactive molecules may be considered alternative therapeutic strategies to restore GBA homeostasis and protect against neurodegeneration [198,199].

Overall, the microbiota composition can be adapted to the quality of the diet, and this impacts brainstem-controlled food intake and weight gain. Metabolites and bacterial signals are sensed by immune and nervous cells, and when pro-inflammatory products are increased, vagal afferent pathway signaling is altered, and the vagal innervation of central nervous system is compromised. Microbiota restoration is sufficient to drive vagal remodeling and tissue homeostasis and to regulate intake.

## 5. Relationship Between Brain, Gut, and White Adiposity Processes

**Figure 7:**
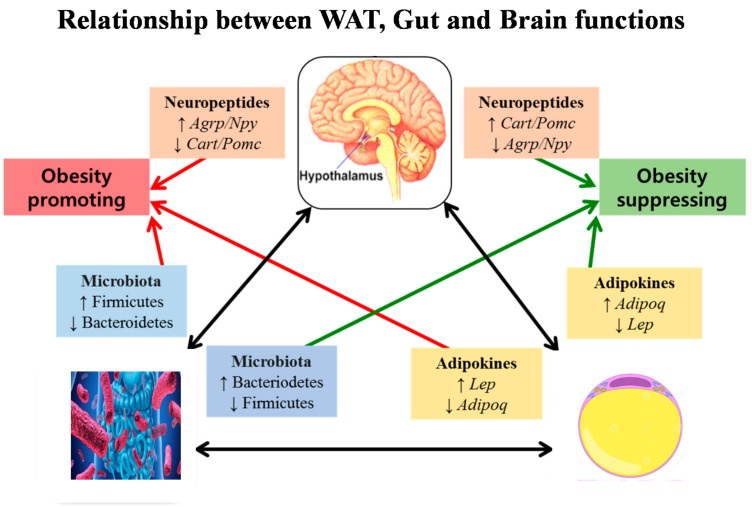
A loop comprising brain and white adipocyte intercommunication in both physiological (in blue) and pathophysiological (in red) conditions. Any disruption of this communication, such as hypertrophic WAT mass expansion, dysbiosis, and altered ANS function, causes metainflammation (obesity/diabetes) and, thereafter, development of neuroinflammation. (AgRP, Agouti-related protein; NPY, neuropeptide Y, CART, cocaine–amphetamine-related transcript; POMC, pro-opiomelanocortin) (adapted from Ansari, et al. [208]).

As has been previously described, there is an intricate crosstalk between the brain, gut, and WAT to accomplish a concerted mechanism to deal with injuries and restore homeostasis and health (Figure 7). Firstly, due to the appearance of endocrine–metabolic dysfunction after WAT inflammation, pro-inflammatory adipokines (WAT-derived) and cytokines (immune-system-derived) increase neuroinflammation by affecting the brain. Indeed, as mentioned, these signals can enter the brain, specifically due to changes in BBB permeability, perpetuating visceral WAT accumulation due to neuronal insulin and leptin sensing being disrupted (IR/LEP-R). As a result, brain dysfunction enhances appetite (incremented orexigenic signals, i.e., NPY and AgRP neuron production) and lowers satiety (decreased anorectic signals, i.e., POMC and CART neuron activities). These mechanisms, combined with the accumulation of the already dysfunctional WAT (excess leptin and decreased adiponectin secretions by large-in-size, IR-resistant white adipocytes), aggravate this picture. Indeed, hypertrophic WAT adipocytes and immune-system-infiltrating cells perpetuate the peripheral inflammatory process as well as neuroinflammation until both (peripheral and brain) dysfunctions are solved. As mentioned, the gut microbiota plays a relevant role in the interrelationship between gut, white adiposity, and brain functions. Indeed, once metainflammation is triggered in an organism (e.g., WAT mass hypertrophic expansion), BBB permeability is modified. As a result, bacteria or viruses may enter the brain, strongly cooperating with neuroinflammation development. Therefore, microbiome perturbations (dysbiosis) then influence both brain and WAT functionalities. All three compartments form part of a pathophysiological vicious cycle of harmful signals. This vicious cycle is summarized in Figure 7, where the concept is clearly represented in both physiological conditions (in blue) and in an obese/diabetic phenotype (in red).

WAT metabolism is also controlled by neuro-immune cells involving adipose-tissue-resident macrophages (ATRMs). Interestingly, a population of white adipose tissue macrophages is colocalized with sympathetic fibers [200]. These cells are known as sympathetic neuro-associated macrophages (SAMs); they are in charge of clearing NE through both solute carrier family 6 member-2 (SLC6A2), a known NE transporter, and monoamine oxidase A (MAOA) activity. SAMs can interact with SNS fibers within WAT, are morphologically distinct from ATRMs, and possess a unique gene expression pattern [200], including an expression of genes related to synaptic signaling, cell–cell adhesion, and neuron development. Unlike the circular morphology of ATRMs, SAMs wrap around SNS fibers and exhibit a stellate morphology. Interestingly, SAMs contain significant amounts of intracellular NE but lack the requisite enzymes for NE synthesis, as has been reported for macrophages [201]. These macrophages are CX3CR1^+^ cells and exhibit a pro-inflammatory shape similar to pro-inflammatory M1 rather than M2 macrophages. Although other macrophages have been shown to express MAOA [200], only SAMs express Slc6a2 [200]. SAMs are thought to act by catching-up with the NE excess after SNS stimulation and thereby metabolizing it. SAMs seem to be recruited to WAT in different obese models (DIO and genetic) and may contribute to white adipocyte hypertrophy through a mechanism of NE over-degradation. Interestingly, ablation of Slc6a2 from SAMs in obese mice leads to obesity rescue by reestablishing NE levels, thereby enhancing lipolysis and energy-expending processes. Remarkably, SAMs were also noticed in human SNS [200]. In most of these studies, CX3CR1^+^ immune cells are implicated in the neuro-immune interaction in WAT. Altogether, these findings suggest that multiple subsets of CX3CR1^+^ macrophages act in concert to maintain energy homeostasis through interactions with SNS innervation of WAT depots.

As expected, due to the proximity between the pancreas, liver, and gut, the origin of nerves innervating the gut is consistent with those innervating the pancreas and liver. Gaskell pioneered the understanding of nerves innervating peripheral tissues, including the gut [201,202]. A pivotal study by Berthoud et al. laid the groundwork for further research in this area [203]. This report concluded that vagal innervation of the gut originates primarily from the gastric branch and celiac branch, while a small contribution from the hepatic branch innervates the distal stomach [203]. Ongoing research is investigating nerve sub-types and their roles in proper gut function. Cholecystokinin receptor (CCKR), a known afferent nerve receptor in the gut [204], increases expression in the nodose ganglia of the vagus in DIO rats [205]. Knockout for *ob-Rb* on sensory nerves in vagal afferents increases body weight gain, indicating inappropriate gut–brain signaling [206]. The latter is crucial evidence for the relevance of gut–brain communication through vagal afferent innervation of the gut and its relationship with adipose tissue accumulation. This finding complements previous data on WAT regarding the presence of the *Ob-Rb* on nerves innervating WAT [33].

Adipose tissue innervation and brain–adipose neural communication offer an exciting area of research. New insights into innervation patterns, neuro-immune interactions, and regulation of nerve plasticity in WAT depots have merged. Other indicators of bi-directional communication between gut-resident nerves and immune cells have recently been studied. In this regard, an interesting role for neurotrophic factors has been implicated in the progression and severity of gut inflammatory responses. One example is nerve growth factor (NGF), secreted by a variety of immune cell types, including mast cells and macrophages, promoting a plethora of signaling pathways and encompassing anti-inflammation and organism survival [207]. The NGF-signaling pathway is linked to knowledge of sensory neuropeptide expression (i.e., substance P) in the rat gut [207]. NGF also promotes the formation of colonic afferent central terminals localized at the dorsal horn of the spinal cord and has been reported to increase visceral nociception in experimental colitis [208].

## 6. Concluding Remarks

Evidence discussed herein supports the significance on the participation of white adiposity and intestinal tract (namely, the gut) dysfunctions as instigators of neuroinflammation due to their interrelationship with the brain (namely, the hypothalamic level). Indeed, malfunction of the WAT–gut axis recorded after injury (e.g., overfeeding, dysbiosis, etc.) seriously compromises hypothalamic function. In turn, overall energy homeostasis disorders and dysmetabolism occur as a result, although peripheral metainflammatory factors released by these tissues at the injured endogenous environment are key signals for neuroinflammation development.

Due to the severe lack of a precise background and information for both clinicians and the general public regarding the fast-growing epidemic condition of insulin-resistance-related pathologies (i.e., metabolic syndrome, obesity, T2DM, and neurodegenerative diseases), early diagnosis and treatment of these pathological conditions are imperative. Even by following WHO recommendations, professional recommendations of following a constant healthy diet (entire fruits, non-manufactured vegetable products, a low carbohydrate ration, a reasonable amount of protein derived from any meat, non-/low-sugar beverages) in combination with sustained physical activity (daily aerobic walking of no less than 40 min) could greatly help to prevent further development of IR-dependent pathologies. Moreover, an early diagnosis of an IR state will help public health systems by reducing future high-cost treatments (e.g., provision of T2DM patients with glucometers and strips, the use of insulin-sensitizing agents, required weight loss surgeries, pharmacological/palliative treatment for neurological disorders, etc.). The preventive early diagnosis of IR-related pathologies will highly improve insulin-resistant patients and their relatives’ quality of life.

In addition to IR states, our body is continuously exposed to dangerous factors, such as malnutrition, dysbiosis, and microbial injury at mucosal sites. To cope with this favorable environment for neuroinflammation development, once metainflammation is established, mucosal intestinal tissues are densely populated with immune cells (the mucosal immune system), and the nervous system is intricately woven into the mucosal immune system. Together, they play a pivotal role in sensing the microbiome composition and orchestrating a proper inflammatory response when dysbiosis is present. The neuro-immune unit is constantly surveilling this complex antigenic context, with the objective of coping with allostasis and, in turn, restoring overall homeostasis through an integrated network of interactions, briefly known as the brain–WAT–gut axis. The microbiota can be considered an additional and key regulator of the function of this axis; indeed, the microbiota is an integral part of this multi-directional axis, thereby influencing tissue physiology. There is compelling evidence that the intervention of the gut microbiota composition may provide new ways of treating metainflammation and neuroinflammation. The use of prebiotics, probiotics, and postbiotics is being explored in pre-clinical and clinical trials.

As a consequence, any individual injury will occur not only in disturbed local tissues (WAT and gut) but also in distant tissues (the brain). Thus, neuroinflammation becomes established until precise reward mechanisms are activated to cope with this detrimental allostasis.

## Abbreviations

Ab	Antibody
Aβ	Amyloid beta
Ach	Acetylcholine
AD	Alzheimer’s disease
AgRP	Aguti-related protein
AKT	Serine/threonine kinase
ALS	Amyotrophic lateral sclerosis
ANS	Autonomic nervous system
Arg1	Arginase 1
ASO	α-Syn-overexpressing
α-Syn	α-Synuclein
ATP	Adenosine triphosphate
ATRM	Adipose tissue-resident macrophage
BBB	Blood–brain barrier
BMP2	Bone morphogenetic protein 2
BMPR	Bone morphogenetic protein receptor
CART	Cocaine–amphetamine-related transcript
CCKR	Cholecystokinin receptor
CGRP	Calcitonin-gene-related peptide
CNS	Central nervous system
CpG ODN	CpG oligodeoxynucleotide
C-RP	C-reactive protein
CSF1	Colony-stimulating factor 1
CX3CL1	Chemokine fractalkine
CX3CR1	Fractalkine receptor 1
DA	Dopamine
DAMP	Damage-associated molecular pattern
DC	Dendritic cells
DIO	Diet-induced obesity
DOPA	Dihydroxy phenyl alanine
ENS	Enteric nervous system
ER	Endoplasmic reticulum
FFA	Free fatty acid
FFAR	Free fatty acid receptor
GBA	Gut–brain axis
GC	Glucocorticoid
GLU	Glucose
GM	Gut microbiota
GR	Glucocorticoid receptor
GRE	Glucocorticoid response element
GSK3β	Glycogen synthase kinase 3β
GVB	Gut–vascular barrier
HD	Huntington’s disease
HFD	High-fat diet
HPA	Hypotalamo-pituitary–adrenal
IAPP	Islet amyloid polypeptide
IAPP	Islet amyloid polypeptide
IBD	Inflammatory bowel disease
IDE	Insulin-degrading enzyme
IFN-γ	Interferon-gamma
IKKβ	Inhibitory kappa beta kinase
IL-1	Interleukin-1
IL-6	Interleukin-6
ILC	Innate lymphoid cell
Ins	Insulin
IR	Insulin resistance
IRS-1	Insulin receptor susbtrate-1
JAK-STAT	Janus kinase–signal transducer and activator of transcription
L2-3	Spinal lumbar segments 2 and 3
LEP-R	Leptin receptor
LPM	Lamina propria macrophage
LPS	Lipopolysaccharide
MAOA	Monoamine oxidase A
microRNA	Micro ribonucleic acid
MM	Muscularis macrophage
MMP	Matrix metalloproteinases
MR	Mineralocorticoid receptor
mTOR	Mechanistic target of rapamycin
ND	Neurodegenerative disease
NE	Norepinephrine
NF	Neuroinflammation
NF-κB	Nuclear factor-kappa B
NGF	Nerve growth factor
NO	Nitric oxide
NPY	Neuropeptide Y
Ob-Rb	Leptin receptor form b
OS	Oxidative stress
PAI-1	Plasminogen activator inhibitor type 1
PAMP	Pathogen-associated molecular pattern
PD	Parkinson’s disease
PI3K	Phosphatidylinositol 3-kinase
PNS	Parasympathetic nervous system
POMC	Pro-opiomelanocortin
PRR	Pattern-recognition receptors
RA	Rheumatoid arthritis
Ramp1	Receptor-activity-modifying protein 1
ROS	Reactive oxygen substances
SAM	Sympathetic neuro-associated macrophage
SCFA	Short-chain fatty acid
SLC6A2	Solute carrier family 6 member-2
SNS	Sympathetic nervous system
SOCS3	Suppressor of cytokine signaling 3
SPF	Specific-pathogen-free
T13	Spinal thoracic segment 13
T2DM	Type 2 diabetes mellitus
Th	T helper lymphocytes
TLR7	Toll-like receptor
TNF-α	Tumor necrosis-alpha
TOX01	Forkhead box protein 01
Trp	Tryptophan
UCP-1	Uncoupling protein-1
VIP	Vasoactive intestinal peptide
WAT	White adipose tissue
WHO	World Health Organization
